# Determinants of visual functions in patients with early and intermediate age-related macular degeneration: the PEONY study

**DOI:** 10.1038/s41433-025-03931-x

**Published:** 2025-07-21

**Authors:** Fangyao Tang, Shruti Chandra, Manjot K. Grewal, Syed Ahmer Raza, Naomi Wijesingha, Livia Faes, Dun Jack Fu, Wei-Shan Tsai, Alicia Lim, Sobha Sivaprasad

**Affiliations:** 1 Institute of Ophthalmology, University College London, London, UK; 2 National Institute for Health Research Biomedical Research Centre, Moorfields Eye Hospital National Health Service Foundation Trust, London, UK

**Keywords:** Macular degeneration, Risk factors

## Abstract

**Background/objectives:**

Although decline in visual functions have been reported in eyes with non-advanced age-related macular degeneration (AMD), it is not known if visual functions in these eyes are influenced by structural changes on optical coherence tomography (OCT). We investigated the association between known OCT changes with photopic and scotopic visual functions.

**Subjects/methods:**

Participants aged 55 years or over with early or intermediate AMD in at least 1 eye, and controls with healthy maculae and were included. Associations between visual functions and retinal structural changes were investigated using linear regression and survival analysis.

**Results:**

We found that the presence of refractile drusen and nascent geographic atrophy (nGA) and were associated with poorer best-corrected visual acuity (BCVA), low luminance VA (LLVA), and increased low luminance deficit (LLD) (*P* < 0.05). In survival analysis, eyes with thicker subfoveal choroidal thickness (SFCT) had a higher hazard rate of rod intercept, suggesting a decreased rod-intercept time (RIT). Eyes with nGA, drusen, refractile drusen, subretinal drusenoid deposits (SDD) have a significantly lower hazard rate of rod intercept (i.e. increased RIT, *P* < 0.05). Among them, thinner SFCT, drusen, and SDD were identified as independent factors associated with an increased RIT in the final multivariable model (*P* < 0.05).

**Conclusions and relevance:**

Given the associations between visual functions with outer retinal layers thickness and presence of established precursors of progression to advanced AMD, our findings serve as a strong foundation for future investigations into the relationships between retinal phenotypes and functional changes.

## Introduction

Advanced forms of age-related macular degeneration (AMD) remain a leading cause of legal blindness globally especially in developed countries [1]. Knowledge of visual function losses that occur before the onset of the advanced forms of the disease is important to design clinical trials on prevention of disease progression. These trials focus on eyes with intermediate AMD defined as presence of large drusen >125 µm and/or extra-foveal retinal pigment epithelial changes. However, some of these visual function changes occur in eyes with no AMD and early AMD. It is therefore valuable to correlate detailed multimodal imaging characteristics of the outer retinal layers with visual functions in eyes with early AMD, intermediate AMD, and those without AMD.

Best-corrected visual acuity (BCVA) is the validated functional primary endpoint accepted by regulatory agencies in clinical trials in AMD [2]. However, changes in BCVA do not parallel disease progression from early or intermediate to late AMD, as BCVA is affected only when the disease involves the fovea [3]. In contrast, low-luminance VA (LLVA) has been shown to correlate with parafoveal retinal sensitivity and patient-reported night vision symptoms in earlier disease stages [4, 5]. Consequently, LLVA deterioration can be observed independently of changes in standard luminance BCVA. Low luminance deficit (LLD) is the difference between standard luminance BCVA and LLVA. Both change in LLVA and LLD have been used as outcome measures for clinical trials [6]. Another measure of visual function is rod mediated dark adaptation (RMDA), which refers to recovery of light sensitivity in a dark environment after exposure to bright light has photobleached a significant proportion of the visual pigments [7]. It can be estimated by rod-intercept time (RIT), the time taken by the rods to recover to an established criterion sensitivity after focal bleaching [8]. Several reports have shown that RIT is more delayed in eyes with subretinal drusenoid deposits (SDDs) and with more severe AMD [9, 10]. There is a paucity of data examining whether these visual functions are influenced by other structural changes in AMD. Such data will provide a solid foundation for future investigations into the relationships between retinal phenotypes and functional changes.

This study therefore aimed to evaluate various visual function measures (BCVA, LLVA, LLD and RIT) across eyes with early AMD, intermediate AMD, and those without any AMD. Specifically, we investigated the influences of retinal structural changes and AMD features on RIT using survival analysis.

## Methods

### Study cohort

This is a secondary analysis of the functional and structural phenotypes in genotypes at varying risk for age-related macular degeneration (PEONY) study, which is a single centre, prospective cohort study conducted at Moorfields Eye Hospital, London, United Kingdom. Inclusion criteria included individuals aged 55 years or over with healthy fundus, or the presence of small drusen or druplets of <63 µm diameter, or those with early or intermediate AMD and Snellen visual acuity of 20/60 or better in at least one eye with media clarity, pupillary dilation, and subject cooperation sufficient for adequate imaging and functional tests. If both eyes of a given participant met the eligibility criteria, the better seeing eye was selected. If the monocular scores were found to be equal, an eye was either chosen randomly or according to the participant preference at the point of consent. Exclusion criteria comprised participants with diabetes; eyes with advanced AMD (neovascular AMD and/or geographic atrophy); eyes with glaucoma; eyes with a refractive error of greater than −6.0 dioptres (D); other eye pathologies that interfere with imaging and visual function examinations (e.g., substantial cataract and corneal opacities); and history of major ocular surgery (including cataract extraction, scleral buckle, any intraocular surgery, etc.) within prior 3 months or anticipated within the next 6 months following enrolment.

### Data collection

**Fig. 1:**
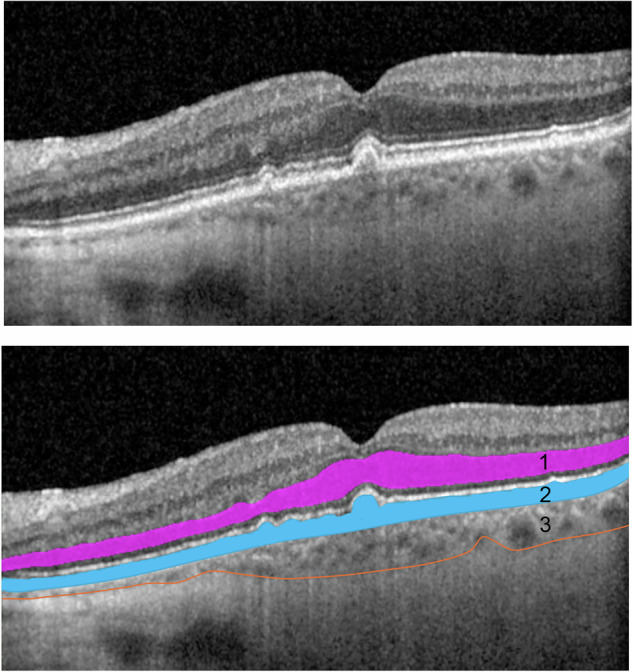
Retinal layer segmentation with detectable layer boundaries in an AMD eye as analysed automatically by the HEYEX software.

A detailed description of the visual function tests and retinal structure evaluations can be found in Supplementary Information 1. Briefly, BCVA was measured at 4 m using the retro-illuminated Early Treatment Diabetic Retinopathy Study chart (ETDRS, Precision Vision, USA) in ETDRS letters. LLVA in ETDRS letters was measured using a 2.0 log neutral density trial lens inserted over the final distance refraction result scores. LLD was calculated by subtracting the LLVA values from the BCVA value. RMDA quantified by RIT was measured using AdaptDx (Lumithera, Poulsbo WA, USA) in a room with lights off with the luminance of 0.01 lux. Retinal pigment epithelium—Bruch membrane (RPE-BM) thickness, outer nuclear layer (ONL) volume, sub-foveal choroidal thickness (SFCT) (Fig. 1), were measured from scans captured by Spectralis Heidelberg Retina Angiograph (HRA) OCT (Heidelberg Engineering GmbH, Heidelberg, Germany). The volumetric images, each consisting of 97 B-scans (posterior pole), were captured in high-speed mode with automated real-time averaging (ART) averaging of 5 frames and quality score over 20. Thicknesses were derived by the built-in software (HEYEX, Heidelberg, Germany). Quantitative fundus autofluorescence (qAF) images were obtained using Spectralis HRA + OCT in the qAF_8_ mode. The Beckman Initiative for Macular Research Classification was used to define the severity of AMD on colour photographs. AMD features were defined as follows. Nascent geographic atrophy (nGA) was defined as the presence of subsidence of the inner plexiform layer and outer plexiform layer, and/or a hyporeflective wedge-shaped band within Henle’s fibre layer on OCT scan [11], with or without the presence of choroidal signal hypertransmission ≥250 μm with an associated zone of retinal pigment epithelium (RPE) attenuation or disruption ≥250 μm [12]. SDD were defined as clear-round or cone-shaped subretinal deposits between external limiting membrane or outer plexiform layers and retinal pigment epithelium (RPE) [13]. Hyperreflective foci (HRF) were defined as hyperreflective dots presented in the retinal layers on OCT scans [14]. Refractile drusen was defined as drusen with deposition of yellowish-white glistening material beneath the retina on colour fundus photograph [15]. Hyporeflective drusenoid lesions (HDL) were defined as bright yellow lesions with defined borders containing glistening dots on colour fundus photographs [16]. Cuticular drusen was defined as multiple yellow or pale, uniform, and round accumulations under the RPE on colour funds photography, and drusen localised beneath the RPE and characterised by RPE elevations on OCT scan [17]. Hypertransmission defects (hyperTDs) were defined as areas of increased focal brightness corresponding to the hyper-transmission of light into the choroid on OCT scan [18]. The presence of any atrophy was defined as presence of hypo-autofluorescence (hypo-AF) on autofluorescence.

### Statistical analysis

All statistical analysis was conducted using R v.4.3.0. Normalities of all continuous variables were examined using Shapiro–Wilk tests and histograms. Continuous and ordinal demographic data were analysed using independent *t*-tests and chi-square test, respectively. Continuous variables were normalised using Z-score normalisation for linear regression analyses, aligning each value in the dataset to a mean of 0 and a standard deviation of 1. This transformation enables comparisons across various types of variables by placing observations on a comparable scale.

Linear regression analyses were performed to determine ocular factors (independent variables) associated with visual function measures (dependent variables). Univariable models were firstly used to determine the associations between visual function with each independent variable. Age, sex, and baseline AMD stage were then included in multivariable models.

It has been shown that a disadvantage of RIT in terms of rod recovery speed is that some eyes exhibit very slow recovery after bleaching, which prevents the RIT measurement from being recorded within the maximum allowed time for the test (usually 20–40 min) [9, 19]. Therefore, these RIT values are recorded as censored data, and are analysed using *t*-test or non-parametric test. However, such approaches may not address the issue of bias arising from the censored data on one hand, or they may be limited by their relative lack of power and so large sample sizes are required. Recently, Higgins et al proposed that ‘time-to-event’ (i.e. survival analysis) could be more appropriate to be applied to RIT as it accommodates for both skewed and censored data and offers higher statistical power [20].

In this study, survival analysis was used to model the RIT adjusted for covariables. Here we applied the approach to depict the cumulative occurrence of the event of interest, which was treated as recovery of sensitivity to the stimulus intensity, within each group. The survival analysis can be used, as the RIT essentially represents the elapsed time until the event of interest is observed [20]. We used Cox proportional hazard model provided in the survival package for R. A hazard ratio (HR) ranged from 0 to 1 indicates a lower probability of reaching a pre-specified threshold (5.0 × 10^−3^ scotopic cd/m^2^) of rod sensitivity recovery following photo bleaching (i.e. an increased RIT), while HR greater than 1 indicates that a greater probability of reaching rod intercept after photo bleach (i.e. a decreased RIT) [21]. Additional multivariable analyses were performed in the control group and the AMD group, respectively. In addition, correlations between RIT and ocular factors in all participants were also examined using Spearman’s correlation model. Adjustment for covariates were performed in partial correlation model. A *P* value ≤ 0.05 was considered as a level of statistical significance.

## Results

The PEONY Study was approved by the London-Chelsea Research Ethics Committee London REC 19/LO/0931. Written informed consent was obtained from all participants and the study followed the tenets of the Declaration of Helsinki.

Table 1 presents the demographics and clinical characteristics of the eligible participants included in analysis (*n* = 225). The mean age (SD) of the group of 49 participants with healthy maculae (stage 1) was 63.57 (5.98) years and 19 (39%) were males. The AMD group consisted of 176 participants had a diagnosis of early AMD (*n* = 46) or intermediate AMD (*n* = 130), with mean age (SD) of 72.95 (8.70) years and 60 (34%) were males.

**Table 1:** Baseline characteristics and comparisons between participants with healthy maculae and those with early or intermediate AMD.

	Participants with healthy maculae (AMD stage 1)	Participants with early or intermediate (AMD stage 2 or 3)	*P*-value
Eyes (*n*)	**49**	176	
Age (years)	63.57 (5.98)	72.95 (8.70)	<0.001
Gender (*n*)			0.661
Male	19 (38.78)	60 (34.10)	
Female	30 (61.22)	116 (65.90)	
BCVA (number of letters)	87.00 (4.91)	81.14 (5.25)	<0.001
LLVA (number of letters)	73.96 (5.56)	66.69 (10.18)	<0.001
LLD (number of letters)	13.04 (4.00)	14.44 (7.93)	0.233
qAF value	243.08 (77.17)	180.13 (79.74)	<0.001
SFCT (µm)	260.06 (80.99)	198.20 (82.88)	<0.001
RPE-BM volume (µm)	0.37 (0.04)	0.47 (0.13)	<0.001
ONL volume (µm)	1.77 (0.23)	1.68 (0.22)	0.007
Presence of nGA (*n*)			0.001
Yes	0 (0.00)	26 (14.77)	
No	74 (100.00)	150 (85.23)	
Presence of hyperTD (*n*)			
Yes	0 (0.00)	58 (32.95)	
No	49 (100.00)	118 (67.05)	
Presence of HDL (*n*)			
Yes	0 (0.00)	27 (15.34)	
No	49 (100.00)	149 (84.66)	
Presence of HRF (*n*)			
Yes	0 (0.00)	65 (36.93)	
No	49 (100.00)	111 (63.07)	
Presence of drusen (*n*)			
Yes	0 (0.00)	176 (100.00)	
No	49 (100.00)	0 (0.00)	
Presence of refractile drusen (*n*)			
Yes	0 (0.00)	8 (4.55)	
No	49 (100.00)	168 (95.45)	
Presence of CD (*n*)			
Yes	0 (0.00)	4 (2.27)	
No	49 (100.00)	172 (97.73)	
Presence of SDD (*n*)			
Yes	0 (0.00)	82 (46.59)	
No	49 (100.00)	94 (53.41)	
Presence of hypo-AF (*n*)			0.125
Yes	1 (2.04)	18 (10.23)	
No	48 (97.96)	158 (89.77)	

Values are *n* (%) or mean (SD).

*BCVA* best corrected visual acuity, *CD* cuticular drusen, *HDL* hyporeflective drusenoid lesions, *HRF* hyperreflective foci, *hyperTD* hypertransmission defect, *hypo-AF* hypo-autofluorescence, *LLD* low-luminance deficits, *LLVA* low-luminance visual acuity, *nGA* nascent geographic atrophy, *ONL* outer nuclear layer, *qAF* quantitative autofluorescence, *RPE-BM* retinal epithelium-Bruch’s membrane, *SDD* subretinal drusenoid deposits, *SFCT* subfoveal choroidal thickness.

Table S1 shows the results of univariable linear regression analyses for BCVA, LLVA, and LLD with recorded ocular factors and AMD features in all participants. Aside from cuticular drusen, all other AMD features considered were associated with lower BCVA and LLVA. Lower SFCT and ONL volume, presence of nGA, refractile drusen, and SDD correlated with greater LLD. Each of the AMD features were considered in a multivariable linear regression model while adjusting for baseline AMD stage, age and sex (Table 2). Statistically significant associations between BCVA and nGA (beta = −0.314, 95% confidence interval (CI): −0.594 to −0.033, *P* = 0.029), hyperTD (beta = −0.283, 95% CI: −0.495 to −0.071, *P* = 0.010), HRF (beta = −0.300, 95% CI: −0.513 to −0.087, *P* = 0.006), drusen (beta = −0.565, 95% CI: −0.809 to −0.321, *P* < 0.001), refractile drusen (beta = −0.635, 95% CI: −1.102 to −0.167, *P* = 0.008), and hypo-AF (beta = −0.480, 95% CI: −0.793 to −0.167, *P* = 0.003) were demonstrated. A similar trend was observed between AMD features and worse LLVA, although a statistically significant association was not observed for HRF. In addition, thicker ONL volume (beta = 0.229, 95% CI: 0.126 to 0.333, *P* < 0.001) was associated with better LLVA. Greater LLD was associated with nGA (beta = 0.545, 95% CI: 0.172 to 0.918, *P* = 0.005), refractile drusen (0.966, 95% CI: 0.331 to 1.601, *P* = 0.003), and SDD (beta = 0.600, 95% CI: 0.358 to 0.843, *P* < 0.001), while increased LLD was associated with thinner SFCT (beta = −0.183, 95% CI: −0.314 to −0.052, *P* = 0.006) and less ONL volume (beta = −0.320, 95% CI: −0.441 to −0.199, *P* < 0.001). In sub-group analysis, although no significant association was found between retinal layer thicknesses and visual functions in participants with healthy maculae (Table S2), thinner ONL thickness was associated with poorer LLVA (beta = 0.284, 95% CI: −0.154 to 0.413, *P* < 0.001) and greater LLD (beta = −0.395, 95% CI: −0.548 to −0.243, *P* < 0.001) participants with early or intermediate AMD (Table S3).

**Table 2:** Multivariable associations between visual functions with ocular factors and AMD features in all participants.

	BCVA^a^	LLVA^a^	LLD^b^
**qAF value (per SD)**	0.142	0.386	0.974
	0.079 (−0.026 to 0.184)	0.048 (−0.060 to 0.155)	−0.002 (−0.127 to 0.123)
**SFCT (per SD)**	0.507	0.114	**0.006**
	−0.034 (−0.133 to 0.066)	0.091 (−0.027 to 0.205)	−**0.183** (−**0.314** to −**0.052**)
**RPE-BM volume (per SD)**	0.077	0.338	0.877
	−0.096 (−0.201 to 0.010)	−0.059 (−0.181 to 0.062)	−0.010 (−0.135 to 0.115)
**ONL volume (per SD)**	0.425	**<0.001**	**<0.001**
	0.038 (−0.056 to 0.132)	**0.229 (0.126** to** −0.333**)	−**0.320** (−**0.441** to −**0.199**)
**Presence of nGA**	**0.029**	**<0.001**	**0.005**
	−**0.314** (−**0.594** to −**0.033**)	−**0.577** (−**0.892** to −**0.262**)	**0.545 (0.172** to **0.918**)
**Presence of hyperTD**	**0.010**	**0.005**	0.078
	−**0.283** (−**0.495** to −**0.071)**	−**0.353** (−**0.595** to −**0.111**)	0.248 (−0.027 to 0.523)
**Presence of HDL**	0.170	0.094	0.194
	−0.186 (−0.455 to 0.083)	−0.265 (−0.575 to 0.045)	0.243 (−0.122 to 0.607)
**Presence of HRF**	**0.006**	0.070	0.710
	−**0.300** (−**0.513** to −**0.087)**	−0.229 (−0.475 to 0.017)	0.051 (−0.219 to 0.332)
**Presence of drusen**	**<0.001**	**0.001**	0.303
	−**0.565** (−**0.809** to −**0.321)**	−**0.396** (−**0.675** to −**0.117**)	0.167 (−0.151 to 0.485)
**Presence of refractile drusen**	**0.008**	**<0.001**	**0.003**
	−**0.635** (−**1.102** to −**0.167)**	**−1.035** (−**1.561** to −**0.583**)	**0.966 (0.331** to −**1.601**)
**Presence of CD**	0.256	0.380	0.747
	−0.382 (−1.040 to 0.276)	−0.338 (−1.092 to 0.416)	0.148 (−0.752 to 1.048)
**Presence of SDD**	0.567	**<0.001**	**<0.001**
	−0.058 (−0.255 to 0.139)	−**0.446** (−**0.663** to −**0.228)**	**0.600 (0.358** to −**0.843**)
**Presence of hypo-AF**	**0.003**	**0.007**	0.185
	−**0.480** (−**0.793** to −**0.167)**	−**0.499** (−**0.859** to −**0.139)**	0.290 (−0.138 to 0.717)

Values are *P*-values, beta values and 95% confidence interval. Values with P < 0.05 are highlighted in bold.

*BCVA* best corrected visual acuity, *CD* cuticular drusen, *HDL* hyporeflective drusenoid lesions, *HRF* hyperreflective foci, *hyperTD* hypertransmission defect, *hypo-AF* hypo-autofluorescence, *LLD* low-luminance deficits, *LLVA* low-luminance visual acuity, *nGA* nascent geographic atrophy, *ONL* outer nuclear layer, *qAF* quantitative autofluorescence, *RPE-BM* retinal epithelium-Bruch’s membrane, *SD* standard deviation, *SDD* subretinal drusenoid deposits, *SFCT* subfoveal choroidal thickness.

^a^Adjusted for age, gender and baseline AMD stage.

^b^Adjusted for age and gender.

Table 3 displays the results of time-event analyses of RIT using Cox proportional hazard model. Sixteen (6.63%) eyes with FER over 33% were excluded. In univariable models considering all participants, eyes with larger RPE-BM volume (HR = 0.737, 95% CI: 0.602 to 0.902, *P* = 0.003) and all AMD features except for cuticular drusen have a lower hazard rate of rod intercept, meaning that the instantaneous probability of reaching rod intercept after photo bleach was lower, suggesting these eyes have an increased RIT. While the eyes with thicker SFCT (HR = 1.513, 95% CI: 1.292 to 1.772, *P* < 0.001) and larger ONL volume (HR = 1.319, 95% CI: 1.112 to 1.564, *P* = 0.001) have a higher hazard rate of rod intercept, meaning that the instantaneous probability of reaching rod intercept after photo bleach was higher, suggesting a decreased RIT. Following adjustment for age, sex, and baseline AMD stage (multivariable model 1), eyes with thicker SFCT (HR = 1.287, 95% CI: 1.083 to 1.530, *P* = 0.004) remained statistically significant predictors of decreased RIT. Conversely, eyes with nGA (HR = 0.260, 95% CI: 0.120 to 0.566, *P* < 0.001), drusen (HR = 0.208, 95% CI: 0.135 to 0.320, *P* < 0.001), refractile drusen (HR = 0.126, 95% CI: 0.017 to 0.904, *P* = 0.039), SDD (HR = 0.157, 95% CI: 0.099 to 0.249, *P* < 0.001), and hypo-AF (HR = 0.362, 95% CI: 0.176 to 0.745, *P* = 0.006) were associated with an increased RIT. Finally, multivariable model 2 included statistically significant variables from model 1 in addition to age, sex, and baseline AMD. Here, eyes with thicker SFCT (HR = 1.229, 95% CI: 1.023 to 1.476, *P* = 0.028) demonstrated a higher hazard rate of rod intercept (i.e. decreased RIT), and those with drusen (HR = 0.421, 95% CI: 0.274 to 0.649, *P* < 0.001), SDD (HR = 0.167, 95% CI: 0.105 to 0.266, *P* < 0.001), have a lower hazard rate of rod intercept (i.e. increased RIT). Additionally, similar correlations between RIT and ocular factors were determined by Spearman’s correlation model (Table S4).

**Table 3:** Relationships between rod intercept time and ocular factors in all participants^a^.

	Univariable			Multivariable model 1^b^			Multivariable model 2^c^		
	*P*-value	Hazard ratio	95% CI	*P*-value	Hazard ratio	95% CI	*P*-value	Hazard ratio	95% CI
**qAF value, pe SD**	0.055	1.172	0.997 to 1.379	0.490	1.066	0.890 to 1.277			
**SFCT, per SD**	<0.001	**1.513**	**1.292** to **1.772**	**0.004**	**1.287**	**1.083** to **1.530**	**0.028**	**1.229**	**1.023** to **1.476**
**RPE-BM volume, per SD**	**0.003**	**0.737**	**0.602** to **0.902**	0.257	0.882	0.710 to 1.096			
**ONL volume, per SD**	**0.001**	**1.319**	**1.112** to **1.564**	0.196	1.173	1.013 to 1.358			
**Presence of nGA**	**<0.001**	**0.209**	**0.098** to **0.447**	**<0.001**	**0.260**	**0.120** to **0.566**	0.139	0.526	0.225 to 1.232
**Presence of hyperTD**	**0.013**	**0.622**	**0.427** to **0.906**	0.609	0.895	0.586 to 1.368			
**Presence of HDL**	**0.023**	**0.527**	**0.305** to **0.914**	0.192	0.687	0.391 to 1.207			
**Presence of HRF**	**0.001**	**0.488**	**0.339** to **0.701**	0.224	0.768	0.502 to 1.175			
**Presence of drusen**	**<0.001**	**0.176**	**0.121** to **0.256**	**<0.001**	**0.208**	**0.135** to **0.320**	**<0.001**	**0.421**	**0.274** to **0.649**
**Presence of refractile drusen**	**0.021**	**0.099**	**0.014** to **0.706**	**0.039**	**0.126**	**0.017** to **0.904**	0.071	0.158	0.021 to 1.167
**Presence of CD**	0.989	1.008	0.321 to 3.162	0.671	1.285	0.404 to 4.082			
**Presence of SDD**	**<0.001**	**0.128**	**0.082** to **0.198**	**<0.001**	**0.157**	**0.099 to 0.249**	**<0.001**	**0.167**	**0.105 to 0.266**
**Presence of hypo-AF**	**0.006**	**0.369**	**0.181** to **0.752**	**0.006**	**0.362**	**0.176** to **0.745**	0.288	0.653	0.297 to 1.434

*CD* cuticular drusen, *HDL* hyporeflective drusenoid lesions, *HRF* hyperreflective foci, *hyperTD* hypertransmission defect, *hypo-AF* hypo-autofluorescence, *nGA* nascent geographic atrophy, *ONL* outer nuclear layer, *qAF* quantitative autofluorescence, *RPE-BM* retinal epithelium-Bruch’s membrane, *SD* standard deviation, *SDD* subretinal drusenoid deposits, *SFCT* subfoveal choroidal thickness. Values with p-values less than 0.05 are highlighted in bold.

^a^Participants with healthy maculae (AMD stage 0 or 1), and participants with early or intermediate (AMD stage 2 or 3) were included.

^b^Adjusted for age, gender and baseline AMD stage.

^c^Adjusted for age, sex, baseline AMD stage and variables with *p* < 0.05 in model 1.

None of the ocular factors were associated with RIT in the group of participants with healthy maculae (Table S5). Instead, in the AMD group, eyes with thicker SFCT and larger ONL volume have a higher hazard rate of rod intercept (i.e. decreased RIT), while eyes with nGA, refractile drusen, SDD, and hypo-AF have a lower hazard rate of rod intercept (i.e. increased RIT) in multivariable model 1 adjusted for age, gender, and baseline AMD stage. Associations between SFCT, nGA, refractile drusen, and SDD with RIT remained significant in multivariable model 2 additionally adjusted for variables with *P* < 0.05 in model 1 (Table S6). Correlations between RIT and ocular factors in the AMD group were also examined by Spearman’s correlation model (Table S7). Significant correlation between ONL volume and RIT was not detected in the multivariable model 1. In the multivariable model 2, nGA was not significantly correlated with RIT.

## Discussion

The key findings of our study are the associations of changes in photopic and scotopic visual functions with volume of outer retinal layers in both controls and early and intermediate AMD. In addition, we report associations between various visual function measures and established precursors of progression to advanced AMD. Similar to other reports, in ascending order, the prevalence of SDD was 47%, HRF 37%, hyperTD 33%, HDL 15%, and nGA 15% in our AMD group (*n* = 176). The other risk factors were of low prevalence that included refractile drusen 5% and cuticular drusen in 2%.

When considering the BCVA, LLVA, and LLD in participants with AMD versus those with healthy maculae, we found that both BCVA and LLVA are lower in participants with early and intermediate AMD. The mean BCVA was 81 ETDRS letters in the AMD group but it was 5.86 letters lower than the group with healthy maculae. As both BCVA and LLVA decreased in parallel by 13–14 letters across the groups, no significant difference in LLD was observed, despite the conflicting evidence regarding the impact of early and intermediate AMD on LLD [22, 23]. To explain visual function changes in eyes with normal maculae based on outer retinal layer thicknesses, we found that a decrease in ONL volume were associated with decreased LLVA, and none of the retinal layer thicknesses influenced BCVA, resulting in greater LLD. These observed decrease in outer retinal volume may be a surrogate of the greater age-related loss of rods compared to cones [24].

When we also consider features of early and intermediate AMD, LLVA was again decreased in eyes with thinner ONL. But the thinning did not affect BCVA suggesting a decline in the numbers of rods. However, the presence of certain precursors of atrophy (nGA and refractile drusen) also resulted in lower BCVA, with differing slopes of decline compared with LLVA resulting in increased LLD. Indeed, the presence of refractile drusen showed the strongest association with greatest beta value. On colour fundus photography, refractile drusen exhibits a topographic distribution across the posterior pole, concentrating in the central macula, and autofluorescence imaging show loss of signal attributable to RPE [25]. On the other hand, histological analyses reveal that the increased light refraction (glistening dots) on the lesions could be related to absence of RPE at the top the drusen, implying loss of RPE. Our findings of worse visual function in eyes with refractile drusen corroborate with the additional RPE loss in this group of patients. However, it should be noted that the small sample size of refractile drusen (*n* = 8) may are likely to yield bias such as model overfitting [26], poor generalisability and wide confidence intervals, making interpretation unreliable. A sample size over 10 is widely advocated as the rule of thumb for multivariable Cox proportional hazard model [27].

Our observation that the presence of nGA is also associated with worse visual functions is consistent with a previous report that microperimetric retinal sensitivity is reduced in areas with nGA [28]. The presence of nGA indicate localised areas of visible disruption of the inner nuclear layer and outer plexiform layer. Break in the external limiting membrane, and disruption of the inner segment ellipsoid and RPE bands were also frequently observed [29]. Our findings that nGA is associated with decreased LLVA and LLD support the possible mechanisms that disturbed functions of the horizontal and amacrine cells in the plexiform layers could contribute to declined visual functions under scotopic conditions [30]. A previous study also reported LLVA impairment and increased level of glial fibrillary acidic protein (GFAP), biomarker of Müller cell activation, in eyes with GA [31], suggesting reactive Müller cell gliosis in the disease. Our observations concur with the concept that gliotic Müller cells in the plexiform layers may disturb retinal glutamate metabolism and ion homoeostasis, resulting in neuronal cell death and consequently visual function loss [32]. The findings of our study support nGA as an intermediate step in the progression towards GA, representing a decline in both structural integrity and visual function. Both BCVA and LLVA were also decreased in eyes with hyperTD but LLD did not increase.

It is well-established that SDD is associated with poorer LLVA than BCVA and explains its association with LLD [23]. It may be that more eyes with nGA had concomitant SDD (73%) compared with hypertransmission and SDD (50%) in this cohort. Our findings highlight the need for considering both retinal thickness and atrophy precursors when explaining losses in visual functions in early and intermediate AMD.

When considering the relation of RIT in healthy maculae, the only significant association was the thinner RPE-BM volume with lower hazard rate of rod intercept, meaning that eyes with thinner RPE-BM membrane have lower instantaneous probability of reaching rod intercept after photo bleach (i.e. increased RIT). Previous findings that both RPE layer thinning and increased RIT were associated with ageing [10, 33], indicating the substantial effect of ageing on disturbance in the visual cycle and impaired RPE function.

The associations between lower hazard rate of rod intercept (i.e. increased RIT) and AMD in our study provide further confirmation that phenotypes that manifest as thinner choroid, nGA, refractile drusen, and SDD impair scotopic function. The association of increased RIT and SDD is well established [7, 8, 34, 35]. Some studies hypothesise that there is link between impaired visual cycle and SDD, while others explain that the relation is due to lipid accumulation resulted by impaired recycling of cholesterol [36]. Our finding that increased RIT is also associated with thinner ONL in eyes with AMD, which contains the cell bodies and nuclei of photoreceptor cells, further highlights the critical role of loss of photoreceptors and the formation of SDD [34–37].

Our observation that SFCT is associated with LLD and RIT may represent another mechanism by which decreased retinoid availability impairs dark adaptation in eyes with early and intermediate AMD. Age-related and early AMD-related changes in the choriocapillaris, such as reduced diameter, density, and thickness, compromise the efficiency of extravasation and uptake of circulating vitamin A complexes through membrane specialisations of the choriocapillary endothelium in the classic visual cycle, leading to extracellular deposits and RMDA deficits [37–39]. SDD have been correlated with choroidal vascular abnormalities supported by both histopathologic and imaging studies [9, 40, 41]. Our findings that show an association between presence of SDD and SFCT thinning with increased RIT align with a cross-sectional study by Flamendorf et al, that found that eyes with SDD exhibit increased RIT and thinning of SFCT [9]. These findings further support the potential mechanisms related to choroidal abnormalities and pathologic systemic vascular ageing process. However, the associations of SFCT thinning and presence of SDD with increased RIT in our study were independent of each other, suggesting SDD and choroidal abnormalities exert independent effects on RMDA deficits.

In summary, our findings suggest that the RMDA deficit may result from a combination of photoreceptor cell loss or degeneration, physical obstruction of essential molecule transport due to SDD, and choroidal vascular abnormalities. In addition, SDD is thought to be associated with cardiovascular disease [42], pointing towards the need for lifestyle changes in people with increased RIT, irrespective of the health of the maculae.

We analysed RIT data using a survival model. Addressing potential bias arising from censored data is a key feature of survival analysis [43]. The investigation by Higgins et al compared the statistical power offered by generalised linear model (GLM), *t*-test, and survival model for censored data. They found that survival model required the smallest sample size to achieve 80% power at an *α*  =  0.05 level, and that the estimated effect was less affected by data censoring in the survival model compared to the other two methods. This provides a significant practical advantage in studies where RIT in AMD patients may exceed the maximum time of the test, offering accurate estimates and correspondent CI [20]. Our finding that significant associations between ONL volume and nGA with RIT in eyes with AMD was only detected by survival analysis but not Spearman’s correlation analysis, highlighted the advantage of considering survival analysis for RIT. Furthermore, the association between choroidal thickness and RIT has been investigated by previous studies [23, 44, 45]. Two of them with sample sizes of fewer than 50 eyes, found no significant association using GLM and Spearman’s correlation analysis. Only one study involving 366 eyes, did demonstrate a significant association between the two measures using Spearman’s correlation analysis. Our investigation, using the Cox proportional hazards model with enhanced statistical power and a superior ability to handle censoring bias through its partial likelihood approach, further supports the existence of a genuine association between choroidal thickness and RIT.

Our study had several strengths, including standardised image acquisition and grading protocols, a broad range of visual function tests and advanced retinal imaging allowing for a detailed exploration of the interaction between retinal structures and functions, and applying survival analysis addressing issue of bias on censored data. However, we also acknowledge the limitations of this study. First, it is a cross-sectional analysis, and longitudinal data are required to evaluate the utility of scotopic functions as potential outcome measures for clinical trials. Second, there were statistically significant age differences between the healthy and AMD groups. Yet, we addressed this limitation by excluding participants with healthy maculae aged less than 55 years old and using age-corrected analyses. Third, the Beckman Initiative for Macular Research Classification was designed for clinical use but not for research use. Previous studies compared Beckman Classification Scale to AREDS 9-step in the same eyes and showed that Beckman Classification Scale depletes the early AMD category by moving up 42% of early to intermediate. It may impact the categorisation of subjects and influence the interpretation of study outcomes [46, 47]. Fourth, lens status data are lacking. In phakic eyes, the presence of cataracts may lead to light scatter and reduced visual acuity. Conversely, visual function test results may be enhanced in pseudophakic eyes after cataract removal and intraocular lens implantation. Finally, microperimetry was not included in our study. Given our focus on subtle functional changes, absence of microperimetry limits the topographic linkage between structural abnormalities with localised visual function loss.

## Conclusion

This study demonstrates associations between photopic and scotopic visual acuity with outer retinal thickness and AMD features. Specifically, we identified the independent effects of SFCT and AMD features on RMDA by using survival analysis. Our findings serve as a strong foundation for future investigations into the relationships between retinal phenotypes and functional changes. Furthermore, visual function loss in eyes with healthy maculae offers an opportunity to differentiate ageing changes from those related to AMD.

Supplementary material is available at Eye’s website.

## Summary

### What was known before

- Visual function losses may occur before the onset of the advanced forms of age-related macular degeneration (AMD).
- It is therefore valuable to correlate detailed multimodal imaging characteristics of the outer retinal layers with visual functions in eyes with early AMD, intermediate AMD, and those without AMD.

### What this study adds

- Survival model can be applied to analyse rod-intercept time values.
- Visual function changes are associated with volume of outer retinal layers, and precursors of AMD progression.

## Supplementary information

Supplementary information

Supplementary Tables

## Data Availability

The data collected for the current study, including individual patient data and a data dictionary defining each field in the data set, will not be made available to others.
