# Supplementary material for: Determinants of visual functions in patients with early and intermediate age-related macular degeneration: the PEONY study
Source: Eye (Lond). 2025 Jul 21;39(14):2686–93. doi: 10.1038/s41433-025-03931-x (PMC12446449; doi:10.1038/s41433-025-03931-x)
Supplement: Supplementary file 1 — Supplementary information [file 41433_2025_3931_MOESM1_ESM.docx]

**Data collection**

Demography and clinical features

Age at recruitment, sex and lens status were recorded.

Distance visual acuity

BCVA was measured at 4 m using the retro-illuminated Early Treatment Diabetic Retinopathy Study chart (ETDRS, Precision Vision, USA) in ETDRS letters.

Low-luminance visual acuity and low luminance deficit

LLVA in ETDRS letters was measured using a 2.0 log neutral density trial lens inserted over the final distance refraction result scores. LLD was calculated by subtracting the LLVA values from the BCVA value.

Rod-mediated dark adaptation

RMDA quantified by RIT was measured using AdaptDx, Lumithera, Poulsbo WA USA).(1) Testing was performed in a room with lights off with the luminance of 0.01 lux. Testing commenced with the study eye bleached using exposure to a flash (duration of 0.25 milliseconds at 58 000 scotopic cd/m^2^ seconds, equivalent to a bleaching level of approximately 83% for rods). This bleaching light subtends 4° and was centred at 5° inferiorly in the vertical meridian, thus projected superiorly to the fovea. This was also the location of the test target. The stimulus for the threshold measurement was a diameter of 2°, a 500 nm circular target that began 15 seconds after the offset of bleaching. The procedure continued in intervals (30 seconds), with a break between each interval (15 seconds) until either the RIT was met, or the test protocol ended (20 minutes), whichever occurred first.(2) The speed of RMDA was characterized by the RIT, defined by the duration required for sensitivity to recover to a value of 5.0 × 10^−3^ scotopic cd/m^2^ (3.0 log units of stimulus attenuation).(3) Eyes with fixation error rate (FER) over 33% were excluded.

Grading of the macular phenotypes

Grading of AMD stages on colour photographs was based on the Beckman Initiative for Macular Research Classification.(4) The stages included: (1) no evidence of AMD or normal aging was defined as healthy maculae or the presence of small drusen or druplets of < 63 µm diameter; (2) early AMD included eyes with a medium drusen, between 63 µm and 124 µm in diameter, with no pigmentary changes; (3) intermediate AMD (iAMD) was defined as eyes with a drusen diameter of ≥ 125 µm or medium drusen with pigmentary changes; and (4) eyes with geographic atrophy. Morphological features on spectral domain optical coherence tomography (SD-OCT) scans were captured on Spectralis Heidelberg Retina Angiograph (HRA) OCT (Heidelberg Engineering GmbH, Heidelberg, Germany). AMD features were defined as follows. Nascent geographic atrophy (nGA) was defined as the presence of subsidence of the the inner plexiform layer (INL) and outer plexiform layer (OPL), and/or a hyporeflective wedge-shaped band within Henle’s fiber laye on OCT scan,(5) with or without the presence of choroidal signal hypertransmission ≥ 250 μm with an associated zone of retinal pigment epithelium (RPE) attenuation or disruption ≥ 250 μm.(6) hypertransmission defects (hyperTDs) were defined as areas of increased focal brightness corresponding to the hyper-transmission of light into the choroid.(7) Hyporeflective drusenoid lesions (HDL) were defined as bright yellow lesions with defined borders containing glistening dots on color fundus photographs.(8) Hyperreflective foci (HRF) were defined as hyperreflective dots presented in the retinal layers on OCT scans.(9) Refractile drusen was defined as drusen with deposition of yellowish-white glistening material beneath the retina on color fundus photograph,(10) Cuticular drusen was defined as multiple yellow or pale, uniform, and round accumulations under the RPE on color funds photography, and drusen localized beneath the RPE and characterized by RPE elevations on OCT scan.(11) SDDs were defined as clear-round or cone-shaped subretinal deposits between external limiting membrane or outer plexiform layers and retinal pigment epithelium (RPE).(12) The presence of any atrophy was defined as presence of hypo-autofluorescence (hypo-AF) on autofluorescence. Our image grading was performed by well-trained retinal specialists with over 30 years of clinical experience.

Macular thickness measurements

The volumetric images, each consisting of 97 B-scans (posterior pole), were captured in high-speed mode with automated real-time averaging (ART) averaging of 5 frames and quality score over 20. The retinal thickness map was derived by the built-in software (HEYEX, Heidelberg, Germany), displaying numeric averages of the measurements for each of the nine ETDRS regions.(13) Retinal pigment epithelium - Bruch membrane (RPE-BM) and outer nuclear layer (ONL) volume, and sub-foveal choroidal thickness (SFCT) within the ETDRS grid were measured by the built-in software after manual correction of segmentation. The RPE-BM was defined as hyperreflective signal that belongs to the 4^th^ of the 4 outer retinal hyperreflective bands(14) or 14^th^ retinal layer(15), and the ONL was defined as the 8^th^ retinal layer(15) typically found on commercial SD-OCT devices. The choroid was defined as the zone beneath the RPE-BM with a marked change in texture in which large circular or ovoid profiles abut a homogenous region of variable reflectivity(15) (**Figure 1**).

Quantitative fundus autofluorescence

Quantitative fundus autofluorescence (qAF) image acquisition and analysis has previously been reported.(16) Briefly, images were obtained using Spectralis HRA + OCT in the qAF_8_ mode with room lights turned off. Each image was acquired in high-speed video mode, at least 2 images were recorded, each being 12 frames. The qAF images were analysed using the HEYEX software. The qAF values were measured from the qAF segments, which refer to the middle ring of the Delori grid centred on the fovea that divides the area between 9° to 11° eccentricity from the fovea into eight segments. Vessels were automatically excluded from the analysis by the software. The threshold setting was manually adjusted if necessary. Phakic eyes were subsequently corrected for normative age-related optical media density.(17, 18) No age-adjustment for ocular media absorption was applied for pseudophakic eyes.(18)

**References:**

1. Jackson GR, Edwards JG. A short-duration dark adaptation protocol for assessment of age-related maculopathy. J Ocul Biol Dis Infor. 2008;1(1):7-11.

2. Higgins BE, Montesano G, Crabb DP, Naskas TT, Graham KW, Chakravarthy U, et al. Assessment of the Classification of Age-Related Macular Degeneration Severity from the Northern Ireland Sensory Ageing Study Using a Measure of Dark Adaptation. Ophthalmol Sci. 2022;2(4):100204.

3. Jackson GR, Scott IU, Kim IK, Quillen DA, Iannaccone A, Edwards JG. Diagnostic sensitivity and specificity of dark adaptometry for detection of age-related macular degeneration. Invest Ophthalmol Vis Sci. 2014;55(3):1427-31.

4. Ferris FL, 3rd, Wilkinson CP, Bird A, Chakravarthy U, Chew E, Csaky K, et al. Clinical classification of age-related macular degeneration. Ophthalmology. 2013;120(4):844-51.

5. Wu Z, Luu CD, Ayton LN, Goh JK, Lucci LM, Hubbard WC, et al. Optical coherence tomography-defined changes preceding the development of drusen-associated atrophy in age-related macular degeneration. Ophthalmology. 2014;121(12):2415-22.

6. Sadda SR, Guymer R, Holz FG, Schmitz-Valckenberg S, Curcio CA, Bird AC, et al. Consensus Definition for Atrophy Associated with Age-Related Macular Degeneration on OCT: Classification of Atrophy Report 3. Ophthalmology. 2018;125(4):537-48.

7. Laiginhas R, Shi Y, Shen M, Jiang X, Feuer W, Gregori G, et al. Persistent Hypertransmission Defects Detected on En Face Swept Source Optical Computed Tomography Images Predict the Formation of Geographic Atrophy in Age-Related Macular Degeneration. Am J Ophthalmol. 2022;237:58-70.

8. Goh KL, Abbott CJ, Hadoux X, Jannaud M, Hodgson LAB, van Wijngaarden P, et al. Hyporeflective Cores within Drusen: Association with Progression of Age-Related Macular Degeneration and Impact on Visual Sensitivity. Ophthalmol Retina. 2022;6(4):284-90.

9. Bolz M, Schmidt-Erfurth U, Deak G, Mylonas G, Kriechbaum K, Scholda C. Optical Coherence Tomographic Hyperreflective Foci: A Morphologic Sign of Lipid Extravasation in Diabetic Macular Edema. Ophthalmology. 2009;116(5):914-20.

10. Oishi A, Thiele S, Nadal J, Oishi M, Fleckenstein M, Schmid M, et al. Prevalence, Natural Course, and Prognostic Role of Refractile Drusen in Age-Related Macular Degeneration. Investigative Ophthalmology & Visual Science. 2017;58(4):2198-206.

11. Balaratnasingam C, Cherepanoff S, Dolz-Marco R, Killingsworth M, Chen FK, Mendis R, et al. Cuticular Drusen: Clinical Phenotypes and Natural History Defined Using Multimodal Imaging. Ophthalmology. 2018;125(1):100-18.

12. Tan R, Guymer RH, Luu CD. Subretinal Drusenoid Deposits and the Loss of Rod Function in Intermediate Age-Related Macular Degeneration. Invest Ophthalmol Vis Sci. 2018;59(10):4154-61.

13. Early Treatment Diabetic Retinopathy Study design and baseline patient characteristics. ETDRS report number 7. Ophthalmology. 1991;98(5 Suppl):741-56.

14. Spaide RF, Curcio CA. Anatomical correlates to the bands seen in the outer retina by optical coherence tomography: literature review and model. Retina. 2011;31(8):1609-19.

15. Staurenghi G, Sadda S, Chakravarthy U, Spaide RF. Proposed lexicon for anatomic landmarks in normal posterior segment spectral-domain optical coherence tomography: the IN•OCT consensus. Ophthalmology. 2014;121(8):1572-8.

16. Chandra S, Grewal MK, Gurudas S, Sondh R, Bird A, Jeffery G, et al. Quantitative Autofluorescence in Non-Neovascular Age Related Macular Degeneration. Biomedicines. 2023;11(2).

17. Greenberg JP, Duncker T, Woods RL, Smith RT, Sparrow JR, Delori FC. Quantitative fundus autofluorescence in healthy eyes. Invest Ophthalmol Vis Sci. 2013;54(8):5684-93.

18. van de Kraats J, van Norren D. Optical density of the aging human ocular media in the visible and the UV. J Opt Soc Am A Opt Image Sci Vis. 2007;24(7):1842-57.
