# Supplementary material for: Determinants of visual functions in patients with early and intermediate age-related macular degeneration: the PEONY study
Source: Eye (Lond). 2025 Jul 21;39(14):2686–93. doi: 10.1038/s41433-025-03931-x (PMC12446449; doi:10.1038/s41433-025-03931-x)
Supplement: Supplementary file 2 — Supplementary Tables [file 41433_2025_3931_MOESM2_ESM.docx]

| **Table S1**: Univariate associations between demographic and ocular factors with visual functions in all participants. | | | |  |
| --- | --- | --- | --- | --- |
|  | **BCVA** | **LLVA** | **LLD** |  |
| **Age (per SD)** | ***<0.001*** | ***<0.001*** | 0.518 |  |
| **Gender (female)** | ***0.012*** | 0.401 | 0.477 |  |
| **Baseline AMD stage (2 vs.1)** | ***<0.001*** | ***0.001*** | 0.086 |  |
| **Baseline AMD stage (3 vs.1)** | ***<0.001*** | ***<0.001*** | 0.417 |  |
| **qAF value (per SD)** | ***0.006*** | 0.062 | 0.807 |  |
| **SFCT (per SD)** | ***0.009*** | ***<0.001*** | ***0.006*** |  |
| **RPE-BM volume (per SD)** | ***<0.001*** | ***0.028*** | 0.888 |  |
| **ONL volume (per SD)** | ***0.017*** | ***<0.001*** | ***<0.001*** |  |
| **Presence of nGA** | ***<0.001*** | ***<0.001*** | ***0.004*** |  |
| **Presence of hyperTD** | ***<0.001*** | ***<0.001*** | 0.075 |  |
| **Presence of HDL** | ***0.018*** | ***0.008*** | 0.192 |  |
| **Presence of HRF** | ***<0.001*** | ***<0.001*** | 0.628 |  |
| **Presence of drusen** | ***<0.001*** | ***<0.001*** | 0.233 |  |
| **Presence of refractile drusen** | ***<0.001*** | ***<0.001*** | ***0.003*** |  |
| **Presence of CD** | 0.141 | 0.210 | 0.706 |  |
| **Presence of SDD** | ***<0.001*** | ***<0.001*** | ***<0.001*** |  |
| **Presence of hypo-AF** | ***<0.001*** | ***0.001*** | 0.184 |  |
| Values are P-values. |  |  |  |  |
| BCVA: best corrected visual acuity; CD: cuticular drusen; HDL: hyporeflective drusenoid lesions; HRF: hyperreflective foci; hyperTD: hypertransmission defect; hypo-AF: hypo-autofluorescence; LLD: low-luminance deficits; LLVA: low-luminance visual acuity; nGA: nascent geographic atrophy; ONL: outer nuclear layer; qAF: quantitative autofluorescence; RIT: rod-intercept time; RPE-BM: retinal epithelium-Bruch's membrane; SD: standard deviation; SDD: subretinal drusenoid deposits; SFCT: subfoveal choroidal thickness. | | | |  |

| **Table S2.** Multivariable associations between visual functions with ocular factors in participants with healthy maculae. | | | |  |  |
| --- | --- | --- | --- | --- | --- |
|  | **BCVA**^a^ | **LLVA**^a^ | **LLD**^a^ | |  |
| **qAF value (per SD)** | 0.604 | 0.667 | 0.275 | |  |
|  | -0.026 (-0.278 – 0.225) | 0.034 (-0.121– 0.190) | -0.075 (-0.187 – 0.352) | |  |
| **SFCT (per SD)** | 0.734 | 0.593 | 0.273 | |  |
|  | -0.037 (-0.249 – 0.175) | 0.038 (-0.101 – 0.177) | 0.090 (-0.249 – 0.069) | |  |
| **RPE-BM volume (per SD)** | 0.881 | 0.944 | 0.791 | |  |
|  | 0.052 (-0.629 – 0.734) | -0.016 (-0.462 – 0.430) | 0.070 (-0.447 – 0.587) | |  |
| **ONL volume (per SD)** | 0.652 | 0.154 | 0.183 | |  |
|  | 0.045 (-0.149 – 0.238) | 0.092 (-0.032 – 0.216) | -0.099 (-0.244 – 0.045) | |  |
| Values are P-values, beta values and 95% confidence interval. | | | |  |  |
| ^a^ Adjusted for age and gender.  BCVA: best corrected visual acuity; LLD: low-luminance deficits; LLVA: low-luminance visual acuity; ONL: outer nuclear layer; qAF: quantitative autofluorescence; RPE-BM: retinal epithelium-Bruch's membrane; SD: standard deviation; SFCT: subfoveal choroidal thickness. | | | |  |  |
|  |  |  |  |  | |
|  |  |  |  |  | |

| **Table S3.** Multivariable associations between visual functions with ocular factors and AMD features in participants with early or intermediate AMD. | | | |  |
| --- | --- | --- | --- | --- |
|  | **BCVA**^a^ | **LLVA**^a^ | **LLD**^b^ |  |
| **qAF value (per SD)** | 0.073 | 0.454 | 0.728 |  |
|  | 0.110 (-0.009 – 0.230) | 0.052 (-0.084 – 0.188) | 0.030 (-0.138 – 0.198) |  |
| **SFCT (per SD)** | 0.562 | 0.141 | ***0.019*** |  |
|  | -0.033 (-0.146 – 0.080) | 0.105 (-0.034 – 0.245) | ***-0.198 (-0.362*** – ***-0.034)*** |  |
| **RPE-BM volume (per SD)** | 0.075 | 0.397 | 0.622 |  |
|  | -0.099 (-0.207 – 0.009) | -0.058 (-0.193 – 0.076) | -0.036 (-0.180 – 0.108) |  |
| **ONL volume (per SD)** | 0.432 | ***< 0.001*** | ***<0.001*** |  |
|  | 0.040 (-0.066 – 0.154) | ***0.284 (0.154*** – ***0.413)*** | ***-0.395 (-0.548*** – ***-0.243)*** |  |
| **Presence of nGA** | ***0.029*** | ***0.001*** | ***0.012*** |  |
|  | ***-0.318 (-0.601* – *-0.036)*** | ***-0.582 (-0.926*** – ***-0.238)*** | ***0.534 (0.123*** – ***0.945)*** |  |
| **Presence of hyperTD** | ***0.010*** | ***0.010*** | 0.162 |  |
|  | ***-0.284 (-0.497*** – ***-0.071)*** | ***-0.352 (-0.616*** – ***-0.088)*** | 0.224 (-0.089 – 0.537) |  |
| **Presence of HDL** | 0.177 | 0.130 | 0.311 |  |
|  | -0.190 (-0.463 – 0.084) | -0.263 (-0.601 – 0.076) | 0.211 (-0.196 – 0.618) |  |
| **Presence of HRF** | ***0.006*** | 0.094 | 0.918 |  |
|  | ***-0.302 (-0.516*** – ***-0.088)*** | -0.231 (-0.499 – 0.038) | 0.016 (-0.291 – 0.324) |  |
| **Presence of refractile drusen** | ***0.008*** | ***0.001*** | ***0.001*** |  |
|  | ***-0.640 (-1.111*** – ***-0.169)*** | ***-1.036 (-1.609*** – ***-0.462)*** | ***0.948 (0.251*** – ***1.645)*** |  |
| **Presence of CD** | 0.264 | 0.417 | 0.804 |  |
|  | -0.380 (1.044 – 0.284) | -0.342 (-1.166 – 0.481) | 0.125 (-0.862 – 1.112) |  |
| **Presence of SDD** | 0.554 | ***< 0.001*** | ***< 0.001*** |  |
|  | -0.060 (-0.259 – 0.139) | ***-0.187 (-0.339*** – ***-0.212)*** | ***0.627 (0.346*** – ***0.908)*** |  |
| **Presence of hypo-AF** | ***0.019*** | ***0.024*** | 0.229 |  |
|  | ***-0.396 (-0.723*** – ***-0.068)*** | ***-0.472 (-0.878*** – ***-0.066)*** | 0.300 (-0.186 – 0.782) |  |
| Values are P-values, beta values and 95% confidence interval. | | | |  |
| ^a^ Adjusted for age, gender and baseline AMD stage; ^b^ adjusted for age and gender. | | | |  |
| BCVA: best corrected visual acuity; CD: cuticular drusen; HDL: hyporeflective drusenoid lesions; HRF: hyperreflective foci; hyperTD: hypertransmission defect; hypo-AF: hypo-autofluorescence; LLD: low-luminance deficits; LLVA: low-luminance visual acuity; nGA: nascent geographic atrophy; ONL: outer nuclear layer; qAF: quantitative autofluorescence; RPE-BM: retinal epithelium-Bruch's membrane; SD: standard deviation; SDD: subretinal drusenoid deposits; SFCT: subfoveal choroidal thickness. | | | |  |
|  |  |  |  |  |
|  |  |  |  |  |
|  |  |  |  |  |
|  |  |  |  |  |

| **Table S4.** Spearman’s correlations between RIT and ocular factors adjusted for age, sex, and baseline AMD stage in all participants. | | | | | | | |  |  |  |
| --- | --- | --- | --- | --- | --- | --- | --- | --- | --- | --- |
|  | | **Univariable** | | **Multivariable model 1^a^** | | | **Multivariable model 2^b^** | | |  |
|  | | **P-value** | **r** | **P-value** | **r** | **P-value** | | **r** | |  |
| **qAF value, pe SD** | | **0.010** | **-0.202** | 0.389 | -0.069 |  | |  | |  |
| **SFCT, per SD** | | **< 0.001** | **-0.333** | **< 0.001** | **-0.247** | ***0.038*** | | ***-0.141*** | |  |
| **RPE-BM volume, per SD** | | **< 0.001** | **0.277** | 0.934 | -0.006 |  | |  | |  |
| **ONL volume, per SD** | | **0.009** | **-0.174** | 0.101 | -0.110 |  | |  | |  |
| **Presence of nGA** | | **< 0.001** | **0.338** | **< 0.001** | **0.240** | 0.197 | | 0.088 | |  |
| **Presence of hyperTD** | | **0.002** | **0.207** | 0.522 | 0.043 |  | |  | |  |
| **Presence of HDL** | | **0.006** | **0.182** | 0.083 | 0.117 |  | |  | |  |
| **Presence of HRF** | | **< 0.001** | **0.257** | 0.341 | 0.064 |  | |  | |  |
| **Presence of drusen** | | **< 0.001** | **0.497** | **< 0.001** | **0.257** | **0.014** | | **0.167** | |  |
| **Presence of refractile drusen** | | **< 0.001** | **0.222** | **0.014** | **0.165** | 0.075 | | 0.121 | |  |
| **Presence of CD** | | 0.938 | 0.005 | 0.511 | -0.044 |  | |  | |  |
| **Presence of SDD** | | **< 0.001** | **0.654** | **< 0.001** | **0.593** | **< 0.001** | | **0.531** | |  |
| **Presence of hypo-AF** | | **< 0.001** | **0.240** | **0.011** | **0.171** | 0.288 | | 0.072 | |  |
| ^a^ Adjusted for age, gender and baseline AMD stage; ^b^ adjusted for age, sex, baseline AMD stage and variables with p < 0.05 in model 1 | | | | | | | | | |  |
| CD: cuticular drusen; HDL: hyporeflective drusenoid lesions; HRF: hyperrefractive foci; hyperTD: hypertransmission defect; hypo-AF: hypo-autofluorescence; nGA: nascent geographic atrophy; ONL: outer nuclear layer; qAF: quantitative autofluorescence; RIT: rod-intercept time; RPE-BM: retinal retinal epithelium-Bruch's membrane; SD: standard deviation; SDD: subretinal drusenoid deposits; SFCT: subfoveal choroidal thickness. | | | | | | | | | |  |
|  |  |  |  |  |  |  |  |  |  |  |
|  |  |  |  |  |  |  |  |  |  |  |
|  |  |  |  |  |  |  |  |  |  |  |

| **Table S5.** Relationships between rod intercept time and ocular factors in participants with healthy maculae. | | | | | | |  |
| --- | --- | --- | --- | --- | --- | --- | --- |
|  | **Univariable** | | | **Multivariable model 1**^a^ | | |  |
|  | **P-value** | **Hazard Ratio** | **95% CI** | **P-value** | **Hazard Ratio** | **95% CI** |  |
| **qAF value, pe SD** | 0.377 | 1.159 | 0.836 – 1.606 | 0.287 | 1.220 | 0.846 – 1.758 |  |
| **SFCT, per SD** | 0.980 | 1.004 | 0.722 – 1.398 | 0.985 | 1.003 | 0.862 – 1.455 |  |
| **RPE-BM volume, per SD** | 0.112 | 2.547 | 0.805 – 8.066 | 0.130 | 2.549 | 0.759 – 8.558 |  |
| **ONL volume, per SD** | 0.117 | 0.793 | 0.593 – 1.060 | 0.100 | 0.767 | 0.559 – 1.052 |  |
| ^a^ Adjusted for age, and sex. | | | | | | |  |
| ONL: outer nuclear layer; qAF: quantitative autofluorescence; RPE-BM: retinal epithelium-Bruch's membrane; SD: standard deviation; SDD: subretinal drusenoid deposits; SFCT: subfoveal choroidal thickness. | | | | | | |  |
|  |  |  |  |  |  |  |  |

| **Table S6.** Relationships between rod intercept time and ocular factors in participants with early and intermediate AMD. | | | | | | | | | | | |  |
| --- | --- | --- | --- | --- | --- | --- | --- | --- | --- | --- | --- | --- |
|  | **Univariable** | | | **Multivariable model 1**^a^ | | | | **Multivariable model 2^b^** | | | |  |
|  | **P-value** | **Hazard Ratio** | **95% CI** | | **P-value** | **Hazard Ratio** | **95% CI** | | **P-value** | **Hazard Ratio** | **95% CI** |  |
| **qAF value, pe SD** | 0.910 | 0.988 | 0.797 – 1.223 | | 0.795 | 1.030 | 0.823 – 1.291 | |  |  |  |  |
| **SFCT, per SD** | ***< 0.001*** | ***1.428*** | ***1.176*** – ***1.734*** | | ***<0.001*** | ***1.396*** | ***1.146*** – ***1.701*** | | ***0.034*** | ***1.286*** | ***1.019*** – ***1.623*** |  |
| **RPE-BM volume, per SD** | 0.259 | 0.891 | 0.729 – 1.089 | | 0.230 | 0.886 | 0.727 – 1.080 | |  |  |  |  |
| **ONL volume, per SD** | ***0.013*** | ***1.308*** | ***1.059*** – ***1.614*** | | ***0.015*** | ***1.311*** | ***1.054*** – ***1.629*** | | 0.908 | 0.986 | 0.778 – 1.249 |  |
| **Presence of nGA** | ***0.001*** | ***0.261*** | ***0.124*** – ***0.573*** | | ***0.001*** | ***0.278*** | ***0.128*** – ***0.601*** | | ***0.035*** | ***0.379*** | ***0.154*** – ***0.935*** |  |
| **Presence of** **hyperTD** | 0.601 | 0.898 | 0.600 – 1.344 | | 0.657 | 0.912 | 0.607 – 1.370 | |  |  |  |  |
| **Presence of HDL** | 0.230 | 0.709 | 0.404 – 1.243 | | 0.188 | 0.685 | 0.390 – 1.203 | |  |  |  |  |
| **Presence of HRF** | 0.221 | 0.780 | 0.524 – 1.161 | | 0.313 | 0.813 | 0.543 – 1.216 | |  |  |  |  |
| **Presence of refractile drusen** | ***0.037*** | ***0.124*** | ***0.017*** – ***0.888*** | | ***0.042*** | ***0.129*** | ***0.018*** – ***0.926*** | | ***0.047*** | ***0.129*** | ***0.017*** – ***0.976*** |  |
| **Presence of CD** | 0.627 | 1.329 | 0.421 – 4.191 | | 0.651 | 1.305 | 0.413 – 4.121 | |  |  |  |  |
| **Presence of SDD** | ***< 0.001*** | ***0.155*** | ***0.098*** – ***0.246*** | | ***< 0.001*** | ***0.157*** | ***0.099*** – ***0.248*** | | ***< 0.001*** | ***0.171*** | ***0.106*** – ***0.273*** |  |
| **Presence of hypo-AF** | ***0.035*** | ***0.439*** | ***0.204*** – ***0.945*** | | ***0.033*** | ***0.435*** | ***0.202*** – ***0.936*** | | 0.796 | 1.125 | 0.461 – 2.746 |  |
| ^a^ Adjusted for age, gender and baseline AMD stage; ^b^ adjusted for age, sex, baseline AMD stage and variables with p < 0.05 in model 1. | | | | | | | | | | | |  |
| CD: cuticular drusen; HDL: hyporeflective drusenoid lesions; HRF: hyperreflective foci; hyperTD: hypertransmission defect; hypo-AF: hypo-autofluorescence; nGA: nascent geographic atrophy; ONL: outer nuclear layer; qAF: quantitative autofluorescence; RPE-BM: retinal epithelium-Bruch's membrane; SD: standard deviation; SDD: subretinal drusenoid deposits; SFCT: subfoveal choroidal thickness. | | | | | | | | | | | |  |
|  |  |  |  |  |  |  |  |  |  |  |  |  |

| **Table S7.** Spearman’s correlations between RIT and ocular factors adjusted for age, sex, and baseline AMD stage in in participants with early and intermediate AMD. | | | | | | | |  |  |
| --- | --- | --- | --- | --- | --- | --- | --- | --- | --- |
|  | | **Univariable** | | **Multivariable model 1^a^** | | | **Multivariable model 2^b^** | | |
|  | | **P-value** | **r** | **P-value** | **r** | **P-value** | | **r** | |
| **qAF value, pe SD** | | 0.852 | -0.017 | 0.740 | -0.031 |  | |  | |
| **SFCT, per SD** | | **< 0.001** | **-0.271** | **< 0.001** | **-0.258** | ***0.010*** | | ***-0.197*** | |
| **RPE-BM volume, per SD** | | 0.111 | 0.120 | 0.086 | 0.131 |  | |  | |
| **ONL volume, per SD** | | **0.030** | **-0.163** | 0.059 | -0.143 |  | |  | |
| **Presence of nGA** | | **< 0.001** | **0.309** | **< 0.001** | **0.296** | 0.119 | | 0.120 | |
| **Presence of hyperTD** | | 0.430 | 0.060 | 0.527 | 0.048 |  | |  | |
| **Presence of HDL** | | 0.173 | 0.103 | 0.153 | 0.109 |  | |  | |
| **Presence of HRF** | | 0.148 | 0.110 | 0.209 | 0.096 |  | |  | |
| **Presence of RD** | | **0.004** | **0.219** | **0.006** | **0.207** | **0.038** | | **0.160** | |
| **Presence of CD** | | 0.695 | -0.030 | 0.693 | -0.030 |  | |  | |
| **Presence of SDD** | | **< 0.001** | **0.625** | **< 0.001** | **0.620** | **< 0.001** | | **0.581** | |
| **Presence of hypo-AF** | | **0.008** | **0.199** | **0.011** | **0.191** | 0.641 | | 0.036 | |
| ^a^ Adjusted for age, gender and baseline AMD stage; ^b^ adjusted for age, sex, baseline AMD stage and variables with p < 0.05 in model 1 | | | | | | | | | |
| CD: cuticular drusen; HDL: hyporeflective drusenoid lesions; HRF: hyperrefractive foci; hyperTD: hypertransmission defect; hypo-AF: hypo-autofluorescence; nGA: nascent geographic atrophy; ONL: outer nuclear layer; qAF: quantitative autofluorescence; RD: reticular drusen; RIT: rod-intercept time; RPE-BM: retinal retinal epithelium-Bruch's membrane; SD: standard deviation; SDD: subretinal drusenoid deposits; SFCT: subfoveal choroidal thickness. | | | | | | | | | |
|  |  |  |  |  |  |  |  |  |  |
|  |  |  |  |  |  |  |  |  |  |
|  |  |  |  |  |  |  |  |  |  |
